# Inclusion of spray dried plasma in diets fed to young pigs increases the ileal digestibility of crude protein and amino acids of other ingredients in the diet

**DOI:** 10.5713/ab.25.0621

**Published:** 2025-10-22

**Authors:** Hannah M. Bailey, Jimena A. Ibagon, Joy M. Campbell, Hans H. Stein

**Affiliations:** 1 Division of Nutritional Sciences, University of Illinois, Urbana, IL, USA; 2 Department of Animal Sciences, University of Illinois, Urbana, IL, USA; 3 APC LLC., Ankeny, IA, USA

**Keywords:** Additivity, Spray Dried Plasma, Standardized Ileal Digestibility, Weaned Pigs

## Abstract

**Objective:**

An experiment was conducted to test the hypothesis that inclusion of spray dried plasma (SDP) increases the apparent ileal digestibility (AID) of starch and the AID and standardized ileal digestibility (SID) of crude protein (CP) and amino acids (AA) from other ingredients in diets for young pigs.

**Methods:**

Thirty weanling barrows (body weight: 9.3±0.6 kg) with a T-cannula in the distal ileum were allotted to a triplicated 10×3 Youden square design with 10 diets and three periods of 7 d. Eight diets with ingredient combinations typically used in the U.S.A. (corn and soybean meal [SBM]), Canada (wheat, barley, SBM, and fermented SBM), the European Union (corn, SBM, wheat, and barley), and Asia (corn, SBM, ground rice, and fermented SBM) were formulated. Four diets contained no SDP, and four diets contained 6% SDP. A diet with SDP as the sole source of AA and a nitrogen-free diet were also included.

**Results:**

An interaction between region and SDP was observed for the AID and SID of CP and most AA. The SDP did not affect AID or SID of AA and CP in U.S.A. or Asian diets, but SDP increased (p<0.05) the AID of CP and the SID of Gly in the European Union diet. In the Canadian diet, SDP increased (p<0.05) the AID of starch and the SID of CP and most AA.

**Conclusion:**

Addition of 6% SDP to diets based on wheat and barley (Canada and European Union diets) may increase the AID of starch and the SID of CP and AA from other ingredients in the diet. Therefore, the SID of CP and AA in diets containing SDP is not always additive in wheat and barley-based diets.

## INTRODUCTION

Weaning is a stressful time for pigs, often resulting in a temporary drop in feed intake, leading to physiological changes in the structure and function of the intestinal tract [1–3]. After weaning, villus atrophy occurs in the small intestine, the activity of brush border digestive enzymes is reduced, and there are greater rates of intestinal protein turnover. Therefore, the capacity for digestion and absorption of amino acids (AA) will be reduced [1,2,4]. Due to these changes and their negative effect on AA digestion and absorption, diets balanced with indispensable AA and formulated with highly digestible protein sources are recommended during the post-weaning period [5]. Animal proteins have greater AA digestibility and contain fewer anti-nutritional factors compared with plant proteins [6], and animal proteins are, therefore, used to provide highly digestible AA to newly weaned pigs.

Spray dried plasma (SDP) is a common animal protein source included at 2% to 8% in diets for newly weaned pigs [7,8]. Inclusion of SDP in diets improves average daily feed intake and average daily gain during the initial 2 wk post-weaning [7], and AA in SDP have a high digestibility when measured in both young pigs [8–10] and growing pigs [11,12]. Addition of SDP to weaned pig diets also improves intestinal barrier function and reduces intestinal inflammation [13]. The improvement in intestinal health may result in an increase in the apparent ileal digestibility (AID) of starch and the AID and standardized ileal digestibility (SID) of crude protein (CP) and AA originating from other ingredients in the diet, but data to demonstrate this are lacking. However, the apparent total tract digestibility of some nutrients and the standardized total tract digestibility of phosphorus increase with the addition of 6% of SDP to the diet, regardless of diet formulation [14].

The SID of AA determined in individual ingredients usually are additive in mixed diets fed to growing pigs [15,16]; however, there are no data demonstrating the additivity of AA in mixed diets containing SDP, and additivity may not always be expected if SDP improves intestinal health, and therefore, increases the SID of AA from other ingredients in the diet. Therefore, an experiment was conducted to test the hypothesis that inclusion of SDP in mixed diets for weanling pigs increases the AID of starch and the AID and SID of CP and AA originating from other ingredients in the diets.

## MATERIALS AND METHODS

### Animals, diets, and feeding

Thirty newly weaned barrows (20±2 d of age) that were the offspring of PIC Camborough females and PIC Line 359 males (Pig Improvement Company) with an initial body weight (BW) of 7.2±0.6 kg were housed in groups of five and fed a phase 1 diet containing 6% SDP for 2 wk post-weaning. After 2 wk, barrows (BW: 9.3±0.6 kg) had a T-cannula installed in the distal ileum [17] and were housed in individual pens (1.2×1.5 m) that were equipped with a self-feeder, a nipple waterer, smooth sides, and fully slatted tribar floors, in an environmentally controlled room. Pigs were fed the phase 1 diet for four or five days for the recovery period after surgery. On day 18 post-weaning, pigs were randomly allotted to a triplicated 10×3 Youden square design [18] with 10 diets and three periods of 7 d. Therefore, there were nine replicate pigs per diet.

SDP (Appetein B) was sourced from APC LLC, and the same batch of SDP was used in all diets containing SDP. Ten experimental diets were formulated (Tables 1, 2). Four diets were formulated with ingredients commonly used in commercial swine diets in the U.S.A. (corn and soybean meal [SBM]), Canada (wheat, barley, SBM, and fermented SBM), the European Union (corn, SBM, wheat, and barley), and Asia (corn, SBM, ground rice, and fermented SBM). These diets did not contain SDP. However, four additional diets were formulated by mixing 94% of the previous four diets with 6% SDP. A diet containing SDP as the sole source of CP and AA that was used to determine the AID and SID of SDP, and a nitrogen-free diet that was used to calculate basal endogenous losses of AA and CP were also formulated. Vitamins and minerals were included in all diets to meet or exceed current nutrient requirement estimates for 7 to 11 kg pigs [19]. All diets, except the phase 1 diet, contained 0.40% chromic oxide as an indigestible marker, and all diets were provided in meal form. At the time of diet mixing, samples of all diets and the main ingredients were collected and used for chemical analysis. Pigs were fed their assigned diets on an ad libitum basis, and water was available at all times. Pig weights were recorded at the beginning of each period and at the conclusion of the experiment.

### Sample collection

Experimental periods were 7 d, with the initial 5 d being the adaptation period, whereas ileal digesta were collected for 9 h (from 08:00 to 17:00 h) on days 6 and 7 following standard procedures [17]. In short, a plastic bag was attached to the cannula barrel, and the digesta flowing into the bag were collected. Bags were removed when filled with ileal digesta, or at least once every 30 minutes, and immediately stored at −20°C to prevent bacterial degradation of the AA in the digesta. At the completion of one experimental period, animals were deprived of feed overnight, and the following morning, the new experimental diet was offered. At the conclusion of the experiment, ileal digesta samples were thawed, mixed within animal and diet, and a sub-sample was lyophilized and finely ground prior to analysis.

### Chemical analysis

All ingredients were analyzed in duplicate for dry matter (method 927.05) [20] and ash (method 942.05) [20], and nitrogen was analyzed by combustion (method 990.03) [20] using a LECO FP628 analyzer (LECO), and CP was calculated as nitrogen×6.25. All ingredients were also analyzed for AA (method 982.30 E [a, b, c]) [20], and gross energy was determined using an isoperibol bomb calorimeter (Model 6400; Parr Instruments) with benzoic acid as the standard for calibration. Acid hydrolyzed ether extract was determined using the acid hydrolysis filter bag technique (Ankom HCl Hydrolysis System; Ankom Technology) followed by crude fat extraction using petroleum ether (AnkomXT15 Extractor; Ankom Technology). Cereal grains were analyzed for starch using the glucoamylase procedure (method 979.10) [20]. Samples of diets and ileal digesta were analyzed for dry matter, CP, AA, and starch as indicated for the ingredients. All diets, except the phase 1 diet, and ileal digesta samples were also analyzed for chromium (method 990.08) [20].

### Calculations

Using equations published by Stein et al [21], values for AID of CP and AA in SDP and AID of CP, AA, and starch in the mixed diets were calculated using the direct procedure. The basal endogenous losses of CP and AA were calculated from pigs fed the nitrogen-free diet, and these data were used to calculate the SID of CP and AA in SDP and the mixed diets. The predicted AID of CP and AA in the four mixed diets containing SDP were calculated according to the following equation [15,16]:


(1) $${\text{AID}}_{\text{p}}=([{\text{AA}}_{\text{SDP}}\times {\text{AID}}_{\text{SDP}}]+[{\text{AA}}_{\text{M}}\times {\text{AID}}_{\text{M}}])/({\text{AA}}_{\text{SDP}}+{\text{AA}}_{\text{M}}),$$

where AID_P_ is the predicted AID for an AA (%) in the mixed diet with SDP; AA_SDP_ and AA_M_ are the concentrations (%) of that AA contributed by SDP and the mixed diet without SDP, respectively, which were calculated by multiplying the concentration of that AA (%) in SDP or the mixed diets without SDP by 6% or 94%, respectively. The AID_SDP_ and AID_M_ are the measured AID (%) for that AA in SDP and the mixed diets without SDP, respectively. The AID of CP and starch in the mixed diets with SDP was predicted using the same equation, and the predicted SID of CP and AA in mixed diets with SDP were also calculated using this equation.

In the calculation for predicted AID and SID values in mixed diets containing SDP, the predicted values for each diet were obtained from mean values of measured AID and SID for AA, CP, or starch in SDP and mixed diets without SDP. The difference between measured and predicted AID and SID values was then calculated by subtracting the predicted AID or SID from the measured AID or SID values determined for the nine replicate pigs per treatment.

### Statistical analysis

At the conclusion of the experiment, normality of residuals was verified, and outliers were identified using the UNIVARIATE and BOXPLOT procedures, respectively (SAS Institute). Outliers were removed if the value deviated from the 1st or 3rd quartiles by more than three times the interquartile range. Data for AID of starch, CP, and AA and SID of CP and AA in the eight diets were analyzed as a 2×4 factorial arrangement of treatments using the MIXED procedure of SAS (SAS Institute), with two levels of SDP and the four regional diets. The pig was the experimental unit for all analyses. The model included dietary concentration of SDP, diet region, and the interaction between SDP and diet region as fixed effects and pig and period as random effects. Treatment means were calculated using the LSMeans statement in SAS, and if significant, means were separated using the PDIFF option in the MIXED procedure. The main effect of period was also analyzed using contrast statements in SAS to determine linear effects of period on the SID of CP and AA over the three periods, with period entered as a class variable with levels 1, 2, and 3. Data from the SDP-only diet and the nitrogen-free diet were used for the calculation of digestibility values of SDP and basal endogenous losses, respectively, and were not included in the factorial statistical model.

Additivity of AID and SID values was analyzed using a t test within each of the four regional diets with added SDP; the null hypothesis that the difference between measured and predicted values for AID and SID was equal to 0 was tested [22]. Statistical differences were established at p≤0.05, whereas 0.05<p≤0.10 was considered a trend.

## RESULTS

The CP in SDP was 80.6% (as-is basis), and the CP in SBM, fermented SBM, soy protein concentrate, and fish meal was 44.2%, 49.2%, 61.8%, and 65.9%, respectively (Table 3). Ground rice contained 77.6% starch (as-is basis), whereas corn, wheat, and barley contained 65.1%, 62.1%, and 60.4% starch, respectively.

The AID of CP in SDP was 80.2% and the AID was greater than 84% for all AA except Cys, Gly, Pro, and Ser (i.e., 89.6%, 86.6%, 84.3%, and 89.2% for Lys, Met, Thr, and Trp, respectively; Table 4). The AID of starch in the SDP diet was greater than 99%. The SID of CP in SDP was 90.4% and the SID was greater than 90% for all AA (i.e., 93.0%, 93.2%, 90.6%, and 93.6% for Lys, Met, Thr, and Trp, respectively).

There was an interaction (p<0.05) between inclusion of SDP and region for the AID of all nutrients except starch, Lys, and Glu (Table 5). The AID of starch in diets with SDP tended to be greater (p<0.10) compared with diets without SDP and the AID of Lys was greater (p<0.05) if SDP was included in the diet. The AID of indispensable AA did not differ between U.S.A. and Asia diets without and with SDP. For the European diet, the AID of Cys tended to be greater (p<0.10) if the diet contained SDP compared with the diet without SDP, and the AID of CP, His, and all dispensable AA, except Ala, Cys, and Glu, was greater (p<0.05) in the diet containing SDP compared with the diet without SDP. For the Canada diet, the AID of CP and all AA, except Lys, Cys, and Glu, was greater (p< 0.05) if SDP was included in the diet, and the AID of Cys tended to be greater (p<0.10) in the Canada diet with SDP compared with the Canada diet without SDP.

An interaction between inclusion of SDP and region for the SID of CP and all AA except Lys and Glu was observed (Table 6). The SID of Lys in diets with SDP was greater (p<0.05) compared with diets without SDP, and the SID of Lys in the Canada diet was less (p<0.05) than in diets from other regions. The SID of Pro was greater (p<0.05) in the U.S.A. diet without SDP compared with SDP, and the SID of Gly was greater (p<0.05) in the European diet containing SDP than in the diet not containing SDP. Except for Pro and Gly, the SID of CP and all other AA did not differ between the U.S.A., European Union, and Asia diets without and with SDP. The SID of CP and all AA, except Lys and Glu, was greater (p<0.05) in the Canada diet with SDP compared with the Canada diet without SDP.

The measured AID of starch was greater (p<0.05) than the predicted value in the U.S.A. and the European Union diet with SDP (Table 7). The measured AID of CP was greater (p<0.05) than predicted in the Asia diet with SDP. The measured AID of Arg was greater (p<0.05) than predicted in the U.S.A diet with SDP. The measured AID of CP, Lys, Glu, Gly, Pro, and Ser was greater (p<0.05) than the predicted values in the European Union diet with SDP, and the measured AID of CP and all AA, except Glu, was greater (p<0.05) than the predicted value in the Canada diet with SDP. The measured SID of CP and AA was consistent with predicted values for the European Union and Asia diets containing SDP, and the measured SID of Trp, Cys, Gly, and Pro was less (p<0.05) than the predicted value for the U.S.A. diet with SDP. The measured SID of CP and AA in the Canada diet was greater (p<0.05) than the predicted SID value for all AA, except Cys and Glu. The SID of CP and all AA linearly increased (p<0.05) from period 1 to period 3 (Table 8).

## DISCUSSION

The chemical composition of all ingredients was generally in agreement with expected values [19]. Diets formulated for the European Union and Canada were based on wheat and barley, which are the cereal grains used as a dietary source of energy in most swine diets in western Canada and northern Europe [23,24]. Barley is the third-most produced grain in Canada after wheat and corn, and approximately 65% of barley is included in animal feed [25]; therefore, barley was included at a greater rate in the Canada diet than in the European Union diet. Wheat is also produced in the U.S.A. and contains more CP and AA than corn [19,26], but because the U.S.A. is the leading global producer of corn, wheat is usually not competitively priced with corn in the U.S.A [24,27]. Therefore, in the current experiment, corn was included as the cereal grain in the U.S.A. diet. The large production of corn and its high concentration of energy makes it a favorable ingredient in swine diets [28], but instead of relying heavily on imports of corn from other countries, rice, the primary cultivated grain in China and south-east Asia, often replaces corn in swine diets in Asia [28,29], and therefore, was included at high concentrations in the Asia diet.

Although diets were formulated to be representative of different regions in the world, the actual raw materials were not purchased in the different countries. Instead, all ingredients were sourced within the United States. It is acknowledged that if certain ingredients produced in other parts of the world have different chemical compositions than ingredients with the same name produced in the United States, diets may not be completely representative of diets actually produced in other countries. However, due to the complications of shipping grain among countries, we chose to source all ingredients in the United States.

The AID of starch in the SDP diet was 99.3%, but SDP does not contain starch and the starch, therefore, originated from the added corn starch in this diet [30]. Values for AID of CP and indispensable AA in SDP were less than some published values [10,11], but in agreement with one of the sources used by Kim et al [31]. Likewise, values for the SID of CP and indispensable AA in SDP were within the range of values previously published [9–11,31].

The AID and SID of AA usually increase as BW increases after weaning up to around 20 kg, resulting in greater AID and SID of AA in pigs greater than 20 kg than in pigs less than 20 kg [4,32,33]. The linear increase in SID of AA that was observed from period 1 to period 3, therefore, is in agreement with published data.

The ileal digestibility of starch varied among diets, regardless of the inclusion of SDP, which is likely a result of the different cereal grains used in the diets, as the AID of starch varies among cereal grains, with rice having the greater digestibility and barley the least [34]. The inclusion of wheat and barley in the Canadian and European Union diets resulted in only minor differences in AID and SID of CP and AA among those diets. However, the AID and SID of most AA were greater in diets formulated without wheat or barley. This reduced digestibility is likely due to the high concentrations of non-starch polysaccharides in wheat and barley, particularly arabinoxylans and β-glucans. These compounds are associated with antinutritive effects, as they increase digesta viscosity and therefore, endogenous AA losses through greater mucin secretion, reducing ileal AA digestibility [34–36].

The tendency for increased AID of starch in the diets when 6% SDP was included, regardless of diet composition, is in agreement with increased digestibility of energy observed by dogs fed kibbles with 1 to 3% SDP [37]. Cereal grains are the main sources of energy in diets due to their high starch concentration and high inclusion rate in diets for swine [38]. Pre-cecal starch digestion is usually between 85% and 95% [34,39]. However, the activity of amylase and maltase, which are needed for starch digestion, is increased in pigs fed a diet containing SDP [40]. Therefore, the greater AID of starch in diets with SDP compared with diets without SDP may be due to an increase in enzymatic activity in the small intestine.

Because the SID of AA in SDP is greater than 90% [9], an improvement in SID of AA in diets containing SDP was expected. SDP of porcine or avian origin, included in a diet fed to weanling pigs, increased the activity of trypsin in the jejunum, which may contribute to the increase in SID of CP and AA in diets containing SDP. However, the activity of trypsin also increases post-weaning as pigs adapt to changes in the concentration of protein, fat, or carbohydrates in solid feed [40]. SDP also has a high concentration of immunoglobulin G that acts as a functional protein in the intestinal tract [41,42], and immunoglobulin G may prevent pathogenic bacteria from colonizing on the mucosal layer of the small intestine [40,41], thereby reducing villus blunting and maintaining the integrity of the mucosa [43]. Pigs fed a diet with SDP have increased villus height, increased villus height-to-crypt depth ratio, and increased villus surface area [41,44], which aids in maintenance of the intestinal barrier post-weaning which is critical for nutrient digestion and absorption [45]. Therefore, SDP may increase the SID of CP and AA by improving the integrity and immunocompetence of the mucosal barrier in young pigs.

The improvement in CP and AA digestibility in the Canada diet with SDP resulted in greater measured AID and SID values than what was predicted, indicating that AID or SID values determined for individual ingredients in the Canada diet are not additive in a mixed diet that contains 6% SDP. Additivity of digestible nutrients in ingredients is a fundamental assumption when formulating diets for swine [15]. AID values obtained for individual ingredients are less additive in a mixed diet compared with SID values for those ingredients [15]. This is due to the underestimation of AID values for low-protein ingredients, such as cereal grains, caused by a greater loss of endogenous AA [15]. Therefore, correcting AID values for basal endogenous losses of AA to calculate SID values results in greater additivity of individual ingredients in a mixed diet [15], which was observed for the European Union diet, but not for the Canada diet containing SDP. As an example, the difference between calculated and measured AID of Lys, Met, Thr, and Trp was between 0 and 2.5 percentage units for the European diet, but between 3.0 and 8.4 percentage units for the Canada diet. Due to the confounding impact of endogenous AA on calculated AID values, values for the SID of AA better describe the AA value of diets because SID values are not impacted by endogenous losses of AA. The Canada diet had greater inclusion of wheat and barley, which are cereal grains with low concentrations of protein, but the greater inclusion rate of these ingredients in the Canada diet may also have resulted in greater concentrations of non-starch polysaccharides [19] and, therefore, anti-nutritional factors further leading to increased endogenous AA losses [21,36]. However, even after correcting AID values for endogenous AA losses, the measured SID values were greater than predicted in the Canada diet indicating that SDP may improve the digestibility of AA in high-fiber ingredients possibly due to improved intestinal health [45]. The interaction between region and SDP that was observed for the SID of most AA is a consequence of the increased SID of AA in the Canada diets containing SDP, which demonstrates that SDP does not influence the SID of AA to the same extent in all types of diets. This observation, therefore, further indicates that some of the barriers to AA digestibility, most likely anti-nutritional factors, in the Canada diet were partly overcome when SDP was added to this diet.

## CONCLUSION

SDP improves the digestibility of CP and AA in diets containing cereals with low AA digestibility. In the Canadian diets, with high inclusions of wheat and barley, measured AID and SID values were greater than predicted values. In contrast, in diets containing less barley and wheat and more corn or rice, SID of CP and AA were not impacted by inclusion of SDP in the diets.

## Figures and Tables

**Table 1:** Ingredient composition of experimental diets

Item (%)	Phase 1	Nitrogen free	SDP	U.S.A.		European Union		Canada		Asia	

				−SDP	+SDP	−SDP	+SDP	−SDP	+SDP	−SDP	+SDP
SDP	6.00	-	18.00	-	6.00	-	6.00	-	6.00	-	6.00
Ground corn	43.16	-	-	55.13	51.83	25.06	23.57	-	-	10.93	10.28
Ground wheat	-	-	-	-	-	20.00	18.80	30.00	28.20	-	-
Ground barley	-	-	-	-	-	15.00	14.10	30.00	28.20	-	-
Ground rice	-	-	-	-	-	-	-	-	-	45.00	42.30
Soybean meal	25.00	-	-	15.00	14.10	9.00	8.46	9.02	8.48	10.00	9.40
Fermented soybean meal	-	-	-	-	-	-	-	7.00	6.58	7.00	6.58
Fish meal	-	-	-	-	-	-	-	-	-	5.00	4.70
Whey powder	20.00	-	-	15.00	14.10	15.00	14.10	15.00	14.10	15.00	14.10
Soy protein concentrate	-	-	-	7.00	6.58	8.00	7.52	-	-	-	-
Corn starch	-	57.45	40.00	-	-	-	-	-	-	-	-
Solka floc	-	4.00	4.00	-	-	-	-	-	-	-	-
Lactose	-	15.00	15.00	-	-	-	-	-	-	-	-
Sucrose	-	15.00	15.00	-	-	-	-	-	-	-	-
Soybean oil	3.10	4.00	4.00	4.00	3.76	4.00	3.76	5.00	4.70	4.00	3.76
Ground limestone	1.20	0.40	1.35	1.00	0.94	1.20	1.13	1.30	1.22	0.90	0.85
Dicalcium phosphate	0.80	2.70	1.20	1.20	1.13	1.00	0.94	0.80	0.75	0.50	0.47
Sodium chloride	0.10	0.40	0.40	0.45	0.42	0.45	0.42	0.45	0.42	0.45	0.42
_L_-Lys HCl	0.29	-	-	0.37	0.35	0.45	0.42	0.58	0.55	0.40	0.38
_DL_-Met	0.15	-	-	0.13	0.12	0.12	0.11	0.13	0.12	0.10	0.09
_L_-Thr	0.05	-	-	0.10	0.09	0.10	0.09	0.10	0.09	0.10	0.09
Magnesium oxide	-	0.10	0.10	-	-	-	-	-	-	-	-
Potassium carbonate	-	0.40	0.40	-	-	-	-	-	-	-	-
Chromic oxide	-	0.40	0.40	0.45	0.42	0.45	0.42	0.45	0.42	0.45	0.42
Vitamin-mineral mix^1)^	0.15	0.15	0.15	0.17	0.16	0.17	0.16	0.17	0.16	0.17	0.16

1) The vitamin-micromineral premix provided the following quantities of vitamins and micro minerals per kilogram of complete diet: vitamin A as retinyl acetate, 11,136 IU; vitamin D_3_ as cholecalciferol, 2,208 IU; vitamin E as DL-alpha tocopheryl acetate, 66 IU; vitamin K as menadione dimethylprimidinol bisulfite, 1.42 mg; thiamin as thiamine mononitrate, 0.24 mg; riboflavin, 6.59 mg; pyridoxine as pyridoxine hydrochloride, 0.24 mg; vitamin B12, 0.03 mg; D-pantothenic acid as D-calcium pantothenate, 23.5 mg; niacin, 44.1 mg; folic acid, 1.59 mg; biotin, 0.44 mg; Cu, 20 mg as copper sulfate and copper chloride; Fe, 126 mg as ferrous sulfate; I, 1.26 mg as ethylenediamine dihydriodide; Mn, 60.2 mg as manganese sulfate; Se, 0.3 mg as sodium selenite and selenium yeast; and Zn, 125.1 mg as zinc sulfate.

SDP, spray dried plasma.

**Table 2:** Analyzed nutrient composition of experimental diets (as-fed basis)

Item (%)	Phase 1	Nitrogen free	SDP	U.S.A.		European Union		Canada		Asia	

				−SDP	+SDP	−SDP	+SDP	−SDP	+SDP	−SDP	+SDP
Dry matter	88.42	92.86	93.68	89.20	88.94	89.04	89.28	89.55	89.36	89.45	89.40
Crude protein	19.73	0.38	12.68	14.54	19.32	14.92	19.26	14.31	18.87	16.25	19.54
Starch	26.73	46.37	36.70	36.47	35.45	35.40	36.06	34.17	35.98	44.72	42.89
Indispensable AA											
Arg	1.30	0.00	0.75	0.96	1.32	0.91	1.16	0.92	1.10	1.12	1.21
His	0.57	0.00	0.41	0.41	0.58	0.38	0.52	0.38	0.49	0.43	0.50
Ile	0.97	0.01	0.44	0.76	0.98	0.71	0.87	0.74	0.83	0.85	0.88
Leu	1.92	0.02	1.33	1.39	1.92	1.25	1.71	1.22	1.57	1.43	1.66
Lys	1.68	0.01	1.27	1.22	1.79	1.10	1.53	1.45	1.51	1.32	1.41
Met	0.43	0.01	0.16	0.34	0.40	0.32	0.34	0.31	0.40	0.50	0.49
Phe	1.07	0.01	0.73	0.77	1.09	0.72	0.98	0.76	0.94	0.83	0.95
Thr	1.05	0.00	0.88	0.76	1.17	0.77	0.97	0.69	0.95	0.80	0.94
Trp	0.33	0.02	0.28	0.21	0.29	0.21	0.29	0.21	0.28	0.22	0.30
Val	1.20	0.01	0.94	0.80	1.24	0.77	1.10	0.81	1.07	0.93	1.11
Total	10.52	0.07	7.19	7.62	10.78	7.14	9.47	7.49	9.14	8.43	9.45
Dispensable AA											
Ala	1.07	0.01	0.70	0.80	1.06	0.73	0.96	0.69	0.87	0.92	1.03
Asp	2.22	0.02	1.39	1.60	2.24	1.45	1.92	1.46	1.78	1.78	1.94
Cys	0.46	0.00	0.49	0.27	0.46	0.29	0.44	0.32	0.46	0.30	0.40
Glu	3.74	0.03	1.97	2.90	3.75	2.94	3.63	3.32	3.65	3.05	3.24
Gly	0.84	0.01	0.49	0.64	0.84	0.64	0.79	0.66	0.76	0.86	0.89
Pro	1.13	0.01	0.67	0.88	1.17	0.92	1.15	1.01	1.16	0.85	0.95
Ser	0.99	0.01	0.78	0.67	0.96	0.61	0.85	0.63	0.82	0.70	0.83
Tyr	0.80	0.01	0.56	0.51	0.77	0.46	0.66	0.49	0.65	0.56	0.67
Total	11.25	0.10	7.05	8.27	11.25	8.04	10.40	8.58	10.15	9.02	9.95
Total AA	21.77	0.17	14.24	15.89	22.03	15.18	19.87	16.07	19.29	17.45	19.40

SDP, spray dried plasma; AA, amino acid.

**Table 3:** Analyzed nutrient composition of ingredients (as-fed basis)

Item	SDP	Ground corn	Soybean meal	Fermented soybean meal	Ground wheat	Ground barley	Soy protein concentrate	Ground rice	Fish meal	Whey powder	Lactose
Dry matter (%)	94.11	86.23	88.13	89.22	87.52	87.65	92.28	87.36	91.79	90.94	94.97
Crude protein (%)	80.56	6.27	44.18	49.15	11.45	9.06	61.76	7.19	65.88	10.67	0.26
Ash (%)	8.75	0.73	5.77	6.89	1.25	2.30	6.37	0.08	19.44	8.04	0.00
GE (kcal/kg)	4,915	3,833	4,202	4,306	3,840	3,772	4,412	3,729	4,379	3,618	3,700
AEE (%)	0.21	2.65	1.84	0.77	1.61	1.70	1.39	1.53	8.97	0.53	0.22
Starch (%)	-	65.09	-	-	62.07	60.35	-	77.55	-	-	-
Indispensable AA (%)											
Arg	4.53	0.36	3.34	3.33	0.51	0.48	4.51	0.51	3.62	0.24	0.00
His	2.41	0.20	1.21	1.28	0.25	0.21	1.64	0.17	1.36	0.20	0.00
Ile	2.49	0.26	2.30	2.53	0.42	0.36	3.18	0.32	2.51	0.68	0.00
Leu	7.49	0.75	3.61	3.93	0.74	0.65	4.94	0.60	4.10	1.09	0.00
Lys	7.24	0.28	2.96	2.85	0.34	0.41	3.97	0.28	4.64	0.90	0.00
Met	0.95	0.14	0.63	0.70	0.17	0.15	0.86	0.18	1.64	0.15	0.00
Phe	4.15	0.34	2.39	2.60	0.51	0.44	3.24	0.38	2.33	0.34	0.00
Thr	5.12	0.25	1.77	1.91	0.30	0.31	2.37	0.24	2.35	0.68	0.00
Trp	1.55	0.07	0.65	0.70	0.13	0.11	0.89	0.08	0.74	0.21	0.02
Val	5.60	0.34	2.40	2.67	0.49	0.50	3.27	0.44	2.87	0.61	0.00
Total	41.53	2.99	21.26	22.50	3.86	3.62	28.87	3.20	26.16	5.10	0.02
Dispensable AA (%)											
Ala	3.95	0.48	2.03	2.25	0.39	0.43	2.74	0.41	3.98	0.53	0.00
Asp	7.90	0.50	5.21	5.59	0.56	0.63	7.08	0.63	5.29	1.10	0.01
Cys	2.69	0.16	0.66	0.73	0.28	0.24	0.89	0.17	0.52	0.26	0.00
Glu	11.29	1.23	8.49	9.13	3.23	1.94	11.53	1.31	7.82	1.85	0.01
Gly	2.77	0.30	2.00	2.24	0.45	0.41	2.67	0.32	4.88	0.22	0.00
Pro	3.84	0.56	2.19	2.41	1.01	0.79	3.03	0.32	2.82	0.58	0.02
Ser	4.90	0.30	1.89	2.03	0.43	0.34	2.51	0.32	1.92	0.42	0.00
Tyr	3.96	0.21	1.70	1.82	0.28	0.23	2.19	0.16	1.76	0.25	0.00
Total	41.30	3.74	24.17	26.20	6.63	5.01	32.64	3.64	28.99	5.21	0.04
Total AA (%)	82.83	6.73	45.43	48.70	10.49	8.63	61.51	6.84	55.15	10.31	0.06

SDP, spray dried plasma; GE, gross energy; AEE, acid hydrolyzed ether extract; AA, amino acids.

**Table 4:** Apparent ileal digestibility (AID) of starch and AID and standardized ileal digestibility (SID) of crude protein and amino acids (AA) in spray dried plasma protein

Item (%)	Spray dried plasma	

	AID	SID
Starch	99.3	-
Crude protein	80.2	90.4
Indispensable AA		
Arg	90.0	96.0
His	87.7	92.5
Ile	84.7	92.3
Leu	89.1	93.2
Lys	89.6	93.0
Met	86.6	93.2
Phe	88.7	93.2
Thr	84.3	90.6
Trp	89.2	93.6
Val	85.1	90.9
Mean	87.7	92.8
Dispensable AA		
Ala	85.9	93.1
Asp	84.4	90.0
Cys	83.1	87.7
Glu	87.0	91.6
Gly	73.6	95.5
Pro	72.3	104.6
Ser	83.5	89.7
Tyr	87.9	93.0
Mean	83.5	92.6
Total AA	85.6	92.7

Data are least squares means of eight (n = 8) observations.

**Table 5:** Apparent ileal digestibility (AID) of starch, crude protein, and amino acids (AA) in experimental diets

Item (%)	U.S.A.		European Union		Canada		Asia		Pooled SEM	p-value		
	
SDP	−	+	−	+	−	+	−	+		SDP	Diet	SDP× Diet
Starch	96.2	97.3	96.3	97.8	97.4	97.1	97.9	98.2	0.57	0.078	0.097	0.385
Crude protein	75.8^ab^	77.1^a^	72.0^b^	78.2^a^	63.6^c^	76.9^ab^	76.6^ab^	80.3^a^	2.17	<0.001	<0.001	0.014
Indispensable AA												
Arg	88.6^abc^	90.3^a^	86.1^c^	88.5^abc^	78.3^d^	86.8^bc^	88.8^abc^	89.4^ab^	1.43	<0.001	<0.001	0.002
His	82.8^ab^	85.1^a^	80.1^b^	83.8^a^	73.5^c^	83.8^a^	83.9^a^	85.5^a^	1.77	<0.001	<0.001	0.002
Ile	83.1^ab^	84.7^a^	80.8^b^	83.0^ab^	73.3^c^	82.4^ab^	84.0^ab^	84.9^a^	1.86	<0.001	<0.001	0.005
Leu	82.9^ab^	85.4^a^	81.1^b^	84.2^ab^	72.9^c^	84.3^ab^	84.2^ab^	86.2^a^	1.91	<0.001	<0.001	0.001
Lys	84.2	87.1	80.9	85.8	78.5	84.0	83.9	84.8	1.43	<0.001	<0.001	0.126
Met	89.1^abc^	88.6^abc^	87.3^bc^	86.8^c^	80.5^d^	89.0^abc^	89.6^ab^	90.3^a^	1.34	0.002	<0.001	<0.001
Phe	83.0^ab^	85.5^a^	80.7^b^	83.8^ab^	72.9^c^	83.4^ab^	83.4^ab^	85.3^a^	1.90	<0.001	<0.001	0.003
Thr	78.0^ab^	81.5^a^	76.8^b^	79.4^ab^	65.3^c^	79.5^ab^	79.4^ab^	81.0^ab^	2.09	<0.001	<0.001	<0.001
Trp	83.5^ab^	83.6^ab^	80.4^b^	83.6^ab^	72.2^c^	83.5^ab^	83.4^ab^	86.8^a^	1.92	<0.001	<0.001	0.005
Val	78.6^ab^	82.5^a^	75.6^b^	79.8^ab^	66.3^c^	80.1^a^	80.9^a^	83.0^a^	2.43	<0.001	<0.001	0.002
Mean	83.2^ab^	85.5^a^	80.8^b^	83.9^ab^	73.6^c^	83.5^ab^	84.1^ab^	85.5^a^	1.77	<0.001	<0.001	0.004
Dispensable AA												
Ala	77.6^bc^	79.7^ab^	74.3^c^	78.1^bc^	63.1^d^	77.7^bc^	80.1^ab^	83.3^a^	2.49	<0.001	<0.001	<0.001
Asp	77.9^ab^	79.6^a^	74.1^b^	78.6^a^	69.2^c^	78.0^ab^	80.4^a^	81.2^a^	1.88	<0.001	<0.001	0.023
Cys	69.4^wxyz^	69.5^xyz^	68.3^yz^	75.0^wx^	63.9^z^	75.8^w^	72.8^wxy^	75.8^w^	3.11	0.002	0.160	0.073
Glu	84.5	82.9	83.3	85.8	80.7	84.5	84.7	85.8	1.88	0.115	0.194	0.184
Gly	67.8^ab^	69.1^a^	61.3^bc^	71.7^a^	57.1^c^	68.8^a^	73.8^a^	73.3^a^	3.01	0.002	<0.001	0.029
Pro	79.0^ab^	79.7^ab^	78.0^bc^	82.1^a^	74.8^c^	82.6^a^	79.7^ab^	80.5^ab^	1.84	0.001	0.664	0.034
Ser	78.8^a^	80.3^a^	74.5^b^	79.7^a^	67.3^c^	78.5^ab^	79.6^a^	81.2^a^	1.94	<0.001	<0.001	0.005
Tyr	81.2^ab^	84.8^a^	77.9^b^	83.3^a^	69.5^c^	82.7^a^	81.4^ab^	84.5^a^	2.25	<0.001	<0.001	0.009
Mean	79.6^ab^	79.9^ab^	76.9^b^	81.2^a^	72.6^c^	80.4^ab^	80.9^ab^	82.2^a^	2.00	<0.001	0.006	0.038
Total AA	81.3^ab^	82.7^a^	78.7^b^	82.5^a^	73.0^c^	81.8^ab^	82.5^a^	83.8^a^	1.87	<0.001	<0.001	0.014

Data are least squares means of nine observations for U.S.A.+SDP treatment (n = 9); eight observations for the European Union+SDP, Canada+SDP, and Asia+SDP treatments (n = 8); and seven observations for U.S.A., the European Union, Canada, and Asia treatments (n = 7).

a–d Means within a row lacking a common superscript letter differ (p<0.05).

w–z Means within a row lacking a common superscript letter differ (0.05<p≤0.10).

SDP, spray dried plasma; SEM, standard error of the mean.

**Table 6:** Standardized ileal digestibility (SID) of crude protein and amino acids (AA) in experimental diets

Item (%)	U.S.A.		European Union		Canada		Asia		Pooled SEM	p-value		
	
SDP	−	+	−	+	−	+	−	+		SDP	Diet	SDP× Diet
Crude protein	84.2^ab^	83.5^ab^	80.3^b^	84.6^ab^	72.2^c^	83.5^ab^	84.2^ab^	86.6^a^	2.17	0.002	0.001	0.016
Indispensable AA												
Arg	93.0^a^	93.5^a^	90.8^a^	92.2^a^	82.9^b^	90.7^a^	92.6^a^	92.9^a^	1.43	0.001	<0.001	0.002
His	87.3^ab^	88.3^a^	85.0^b^	87.3^ab^	78.4^c^	87.6^ab^	88.2^ab^	89.2^a^	1.77	<0.001	<0.001	0.002
Ile	87.3^a^	87.9^a^	85.3^a^	86.7^a^	77.7^b^	86.2^a^	87.8^a^	88.5^a^	1.86	0.002	<0.001	0.004
Leu	86.6^ab^	88.1^ab^	85.2^b^	87.2^ab^	77.1^c^	87.6^ab^	87.8^ab^	89.3^a^	1.91	<0.001	<0.001	0.001
Lys	87.5	89.4	84.6	88.5	81.4	86.7	87.0	87.7	1.43	<0.001	<0.001	0.127
Met	92.0^ab^	91.1^ab^	90.4^ab^	89.7^b^	83.8^c^	91.5^ab^	91.6^ab^	92.3^a^	1.34	0.008	<0.001	<0.001
Phe	87.1^ab^	88.3^ab^	85.0^b^	87.1^ab^	77.0^c^	86.7^ab^	87.2^ab^	88.7^a^	1.90	<0.001	<0.001	0.003
Thr	85.0^a^	86.0^a^	83.7^a^	85.0^a^	73.1^b^	85.2^a^	86.1^a^	86.7^a^	2.09	0.002	<0.001	0.001
Trp	88.9^ab^	87.6^ab^	85.9^b^	87.6^ab^	77.7^c^	87.6^ab^	88.7^ab^	90.6^a^	1.92	0.007	<0.001	0.005
Val	85.0^ab^	86.6^ab^	82.3^b^	84.5^ab^	72.7^c^	84.9^ab^	86.4^ab^	87.6^a^	2.42	<0.001	<0.001	0.002
Mean	87.7^ab^	88.7^ab^	85.6^b^	87.5^ab^	78.2^c^	87.2^ab^	88.2^ab^	89.1^a^	1.77	<0.001	<0.001	0.003
Dispensable AA												
Ala	83.6^ab^	84.2^ab^	80.9^b^	83.1^b^	70.1^c^	83.2^b^	85.4^ab^	87.9^a^	2.49	<0.001	<0.001	0.001
Asp	82.5^ab^	82.9^ab^	79.2^b^	82.5^ab^	74.3^c^	82.2^ab^	84.5^a^	85.0^a^	1.88	0.003	<0.001	0.027
Cys	77.4^abc^	74.2^bc^	75.7^abc^	79.9^ab^	70.6^c^	80.5^a^	80.0^ab^	81.2^a^	3.11	0.069	0.126	0.046
Glu	87.5	85.2	86.2	88.2	83.3	86.9	87.6	88.5	1.88	0.256	0.134	0.141
Gly	83.8^ab^	81.3^ab^	77.3^bc^	84.7^a^	72.7^c^	82.3^ab^	85.7^a^	84.9^a^	3.01	0.051	0.021	0.036
Pro	102.4^ab^	97.3^cd^	100.4^abc^	100.1^bc^	95.3^d^	100.4^abc^	104.0^a^	102.3^ab^	1.84	0.568	0.003	0.004
Ser	85.7^ab^	85.1^ab^	82.0^b^	85.1^ab^	74.6^c^	84.1^ab^	86.1^ab^	86.7^a^	1.94	0.004	<0.001	0.005
Tyr	86.4^ab^	88.3^a^	83.7^b^	87.3^ab^	75.0^c^	86.8^ab^	86.2^ab^	88.6^a^	2.25	<0.001	<0.001	0.009
Mean	86.9^a^	85.4^a^	84.5^a^	87.0^a^	79.7^b^	86.4^a^	87.7^a^	88.3^a^	2.00	0.038	0.008	0.027
Total AA	87.3^ab^	87.0^ab^	85.0^b^	87.3^ab^	79.0^c^	86.8^ab^	88.0^ab^	88.7^a^	1.87	0.005	<0.001	0.011

Data are least squares means of nine observations for U.S.A.+SDP treatment (n = 9); eight observations for the European Union+SDP, Canada+SDP, and Asia+SDP treatments (n = 8); and seven observations for U.S.A., the European Union, Canada, and Asia treatments (n = 7).

Standardized ileal digestibility values were calculated by correcting values for apparent ileal digestibility for the basal ileal endogenous losses. Endogenous losses (g/kg of dry matter intake) AA were as follows: crude protein, 13.80; Arg, 0.48; His, 0.21; Ile, 0.36; Leu, 0.58; Lys, 0.46; Met, 0.11; Phe, 0.35; Thr, 0.60; Trp, 0.13; Val, 0.58; Ala, 0.54; Asp, 0.83; Cys, 0.24; Glu, 0.97; Gly, 1.15; Pro, 2.31; Ser, 0.51; Tyr, 0.30.

a–d Means within a row lacking a common superscript letter differ (p<0.05).

SDP, spray dried plasma; SEM, standard error of the mean.

**Table 7:** Differences between measured and predicted apparent ileal digestibility (AID) values for starch, crude protein, and amino acids (AA) and for standardized ileal digestibility (SID) of crude protein and AA in mixed diets containing spray dried plasma from the U.S.A., European Union, Canada, and Asia

Item (%)	U.S.A.		European Union		Canada		Asia	

	AID	SID	AID	SID	AID	SID	AID	SID
Starch	1.2^*^	-	1.4^*^	-	−0.2	-	0.3	-
Crude protein	0.2	−2.3	4.2^*^	1.9	9.2^*^	6.7^*^	2.7^*^	0.8
Indispensable AA								
Arg	1.4^*^	−0.2	1.7^+^	0.4	5.9^*^	4.8^*^	0.2	−0.6
His	1.0	−0.4	1.7	0.4	6.5^*^	5.5^*^	0.3	−0.4
Ile	1.3	−0.2	1.8	0.4	7.3^*^	6.3^*^	0.5	−0.2
Leu	0.8	−0.3	1.2	0.1	7.1^*^	6.2^*^	0.5	−0.1
Lys	1.4^+^	0.3	2.5^*^	1.6^+^	3.0^*^	2.7^*^	−0.8	−1.1
Met	0.0	−1.0	0.0	−0.8	7.6^*^	6.4^*^	0.8	0.4
Phe	1.0	−0.3	1.3	0.1	6.7^*^	5.8^*^	0.4	−0.2
Thr	1.6	−0.7	0.7	−0.6	8.4^*^	6.7^*^	0.0	−0.9
Trp	−1.7	−2.8^*^	0.6	−0.6	6.0^*^	5.0^*^	1.3	0.2
Val	1.9	−0.2	1.6	−0.1	8.4^*^	7.0^*^	0.6	−0.4
Mean	1.1	−0.3	1.5	0.3	6.4^*^	5.5^*^	0.2	−0.4
Dispensable AA								
Ala	0.1	−1.7	1.2	−0.6	8.9^*^	7.3^*^	1.5^+^	0.6
Asp	0.1	−1.4	2.1^+^	0.7	5.2^*^	4.1^*^	−0.3	−0.9
Cys	−5.2^+^	−7.2^*^	1.6	0.1	5.7^*^	4.4^+^	−1.0	−1.8
Glu	−2.1	−3.1^+^	2.1^*^	1.2	2.9	2.4	0.5	0.0
Gly	−0.2	−5.3^*^	7.9^*^	3.6^+^	8.7^*^	5.3^*^	−0.9	−3.0
Pro	2.2^+^	−5.5^*^	5.6^*^	−1.0	8.5^*^	3.5^*^	2.2	−2.1
Ser	0.0	−1.9	2.4^*^	0.7	6.1^*^	4.8^*^	0.2	−0.7
Tyr	1.4	−0.3	2.1	0.6	7.2^*^	6.0^*^	0.8	−0.1
Mean	−0.6	−2.9^+^	2.9^*^	0.8	5.6^*^	4.0^*^	0.4	−0.7
Total AA	0.3	−1.7	2.2^+^	0.6	6.0^*^	4.7^*^	0.3	−0.6

Difference is calculated by subtracting predicted AID of starch, crude protein, or individual AA from measured value. Likewise, for the difference between predicted value for SID of crude protein or individual AA from measured value.

Data are least squares means of nine observations for U.S.A.+SDP treatment (n = 9); eight observations for the European Union+SDP, Canada+SDP, and Asia+SDP treatments (n = 8); and seven observations for U.S.A., the European Union, Canada, and Asia treatments (n = 7).

* p≤0.05;

+ p≤0.10.

**Table 8:** Effect of period on standardized ileal digestibility (SID) of crude protein and amino acids (AA) in diets fed to weaned pigs

Item (%)	Period^1)^			Pooled SEM	p-value	
	
	1	2	3		Period	Linear
Crude protein	81.9	81.9	86.1	1.88	0.012	0.011
Indispensable AA						
Arg	89.9	91.5	93.5	1.32	0.003	<0.001
His	84.8	87.1	89.5	1.41	<0.001	<0.001
Ile	84.3	86.5	89.2	1.45	<0.001	<0.001
Leu	84.5	86.9	89.4	1.56	<0.001	<0.001
Lys	85.6	87.2	89.2	1.20	0.002	<0.001
Met	88.9	90.5	92.4	1.07	0.003	<0.001
Phe	84.3	86.7	89.2	1.56	<0.001	<0.001
Thr	82.4	84.4	87.2	1.76	0.005	0.001
Trp	85.7	87.3	89.8	1.62	0.013	0.004
Val	81.5	84.3	87.9	1.84	<0.001	<0.001
Mean	85.0	87.1	89.6	1.44	<0.001	<0.001
Dispensable AA						
Ala	80.4	83.5	86.7	2.21	<0.001	<0.001
Asp	80.3	82.8	84.7	1.59	0.004	0.001
Cys	74.7	79.9	81.3	2.00	0.006	0.002
Glu	84.8	87.6	89.3	1.07	0.005	0.001
Gly	80.2	82.8	86.8	2.36	0.005	0.001
Pro	97.8	101.0	103.5	1.46	0.013	0.003
Ser	82.1	84.4	86.6	1.58	0.005	0.001
Tyr	83.4	86.2	89.0	1.79	0.001	<0.001
Mean	83.9	86.8	89.0	1.36	0.001	<0.001
Total AA	84.5	87.0	89.3	1.37	<0.001	<0.001

Data are least squares means of 22 observations for period 1 and 3, and 25 observations for period 2.

1) Pigs had an average body weight of 10.54±2.08, 13.95±3.24, and 18.28±4.06 in periods 1, 2, and 3, respectively.

SEM, standard error of the mean.

## Data Availability

Upon reasonable request, the datasets of this study can be available from the corresponding author.
